# The significance of metabolic disease in degenerative cervical myelopathy: a systematic review

**DOI:** 10.3389/fneur.2024.1301003

**Published:** 2024-02-05

**Authors:** Celine Iswarya Partha Sarathi, Amil Sinha, Amir Rafati Fard, Faheem Bhatti, Tanzil Rujeedawa, Shahzaib Ahmed, Melika Akhbari, Aniqah Bhatti, Aria Nouri, Mark R. Kotter, Benjamin M. Davies, Oliver D. Mowforth

**Affiliations:** ^1^Division of Academic Neurosurgery, Department of Clinical Neurosciences, Addenbrooke’s Hospital, University of Cambridge, Cambridge, United Kingdom; ^2^Division of Neurosurgery, Geneva University Hospitals, University of Geneva, Geneva, Switzerland

**Keywords:** cervical cord, myelopathy, spondylosis, stenosis, ossification posterior longitudinal ligament, metabolism, cardiovascular disease, diabetes

## Abstract

**Introduction:**

Degenerative cervical myelopathy (DCM) is a form of chronic spinal cord injury, with a natural history of potential for progression over time. Whilst driven by mechanical stress on the spinal cord from degenerative and congenital pathology, the neurological phenotype of DCM is likely to be modified by multiple systemic factors. The role of metabolic factors is therefore of interest, particularly given that ischaemia is considered a key pathological mechanism of spinal cord injury. The objective was therefore to synthesise current evidence on the effect of metabolism on DCM susceptibility, severity, and surgical outcomes.

**Methods:**

A systematic review in MEDLINE and Embase was conducted following PRISMA guidelines. Full-text papers in English, with a focus on DCM and metabolism, including diabetes, cardiovascular disease, anaemia, and lipid profile, were eligible for inclusion. Risk of methodological bias was assessed using the Joanna Briggs Institute (JBI) critical assessment tools. Quality assessments were performed using the GRADE assessment tool. Patient demographics, metabolic factors and the relationships between metabolism and spinal cord disease, spinal column disease and post-operative outcomes were assessed.

**Results:**

In total, 8,523 papers were identified, of which 57 met criteria for inclusion in the final analysis. A total of 91% (52/57) of included papers assessed the effects of diabetes in relation to DCM, of which 85% (44/52) reported an association with poor surgical outcomes; 42% of papers (24/57) discussed the association between cardiovascular health and DCM, of which 88% (21/24) reported a significant association. Overall, DCM patients with diabetes or cardiovascular disease experienced greater perioperative morbidity and poorer neurological recovery. They were also more likely to have comorbidities such as obesity and hyperlipidaemia.

**Conclusion:**

Metabolic factors appear to be associated with surgical outcomes in DCM. However, evidence for a more specific role in DCM susceptibility and severity is uncertain. The pathophysiology and natural history of DCM are critical research priorities; the role of metabolism is therefore a key area for future research focus.

**Systematic review registration:**

https://www.crd.york.ac.uk/prospero/, identifier: CRD42021268814.

## Introduction

Degenerative cervical myelopathy (DCM) is a condition of spinal cord dysfunction secondary to mechanical stress from congenital and/or degenerative changes, such as cervical canal stenosis, intervertebral disc herniation, spondylosis, ligament hypertrophy, calcification and ossification (1). It is estimated to affect as many as 2% of adults (2), and given its association with age, incidence is expected to rise as populations age (3). Patients experience a range of disabilities including pain and stiffness, loss of dexterity, bladder and bowel dysfunction (4, 5). This also has significant impacts on those around them and society as a whole (6).

There remain many clinical research uncertainties in DCM, with two of the most fundamental uncertainties, as established by AO Spine RECODE-DCM (7–9), relating to pathobiology and natural history (10–12). For example, whilst spinal cord compression is considered a pathological hallmark of DCM, its detection on MRI is most commonly an incidental finding (3) and does not correlate with disease severity (13). Moreover, disease trajectory, particularly in the early and milder stages of the disease is heterogenous and unpredictable (14, 15). This suggests that additional factors may play a role in influencing spinal cord damage and disease progression (11). Understanding these factors will be important to better inform clinical care.

Age, smoking status and presence of comorbidities have previously been identified as important predictors of outcomes, however their weight on the progression of DCM and response to medical/surgical treatment remains to be further investigated (16). Cardiovascular disease is a prominent global health problem and is closely associated with altered metabolism in the context of obesity and decreased physical activity (17). The World Health Organisation (WHO) defines the metabolic syndrome as a pathological condition characterised by obesity, insulin resistance, hypertension, and hyperlipidaemia. In practise, metabolism encompasses themes such as diabetes, cardiovascular health, and lipids. Both aberrant metabolism and spinal cord hypoperfusion have been proposed as mechanisms of spinal cord injury in DCM (15, 18).

These comorbidities may have further implications for DCM (19). Firstly, degenerative spinal pathology is more prevalent with obesity; for example, the prevalence of degenerative disc disease is higher in those with the metabolic syndrome (20). Secondly, DCM is treated with surgery and the decision to undergo surgery entails a balance of risks and benefits. Surgical patients with metabolic disorders are at higher risk of a range of adverse outcomes, including death, cardiovascular events, stroke, renal failure, surgical site infections, prolonged hospital stays and have a greater need for post-hospitalisation rehabilitation (21). Whilst in more advanced forms of the disease the benefits of surgery are more certain, in milder forms of the disease this may not necessarily be the case, and decision making needs to be tailored to the individual circumstances (22).

The objective of this review was therefore to assess the current evidence for metabolic dysfunction in DCM, and specifically to synthesise evidence relating metabolic dysfunction to disease onset, severity and surgical outcomes.

## Methods

### Study design

A systematic review was conducted with reference to the Preferred Reporting Items for Systematic Reviews and Meta-Analyses (PRISMA) 2020 checklists (Supplementary Data 1) (23). The protocol was registered on PROSPERO (ID: CRD42021268814).

### Eligibility criteria

All primary clinical studies, available in English, considering an aspect of metabolism in the context of DCM were considered eligible for inclusion. Animal studies, case reports, editorials, reviews, opinion articles, corrections and conference papers were excluded. Metabolism was defined as the capability of the body to adapt its endocrine environment according to supply and demand for fuel; such metabolic regulation can be affected by many factors over the course of several years (24). We utilised the WHO definition of metabolic disorders to categorise factors into diabetes and cardiovascular health.

### Search strategy

A search of Embase and MEDLINE using Ovid for all papers published until January 2023 was performed using a modified version of a previously published DCM search strategy (25, 26). The full search terms are outlined in Supplementary Data 2.

### Selection process

Title and abstract screening were completed using Rayyan (Rayyan Systems Inc., Cambridge, MA, United States). Studies were independently screened in duplicate by seven authors (CP, FB, AS, MA, AB, SA and TR); a pilot of 100 records were screened by all reviewers to ensure concordance. Discrepancies were settled by discussion and mutual agreement.

### Data collection

Manual data extraction was completed by seven authors (CP, FB, AS, MA, AB, SA and TR) in Microsoft Excel (Version 16.63, Microsoft 365) using a piloted extraction form (Supplementary Data 3). Details of the study design, cohort demographics, intervention(s), metabolic factor(s) and outcomes were extracted.

### Risk of bias assessment

Risk of methodological bias in individual studies was assessed by two authors (AR and AS) using the Joanna Briggs Institute (JBI) critical assessment tools for cohort or analytical cross-sectional studies depending on study type (Supplementary Data 4) (27).

### Synthesis methods

Due to heterogeneity in study design and data reporting, a qualitative synthesis was performed in accordance with the Synthesis without Meta Analysis (SWiM) guidelines (28). In order to consider the differing implications of metabolic disease on DCM, reported study outcomes were categorised into those relating to spinal column disease (e.g., radiological features of spondylosis), spinal cord disease (e.g., neurological examination, patient-reported outcome measures and recovery rate with treatment) and those relating specifically to the surgical procedure (e.g., adverse events, such as infection). This approach aimed to discern effects of metabolism on spinal cord vulnerability, as opposed to spondylosis or surgical risk (29). Not all studies included outcomes in all three subgroups. Further categorisation for identified metabolic factors was developed to group evidence into diabetes and cardiovascular disease. Studies that considered more than one metabolic factor (i.e., both diabetes and cardiovascular disease) were assigned to both categories. Adverse events of surgery were categorised using the criteria proposed by Tetreault et al. (30).

### Certainty assessment

**Figure 1:**
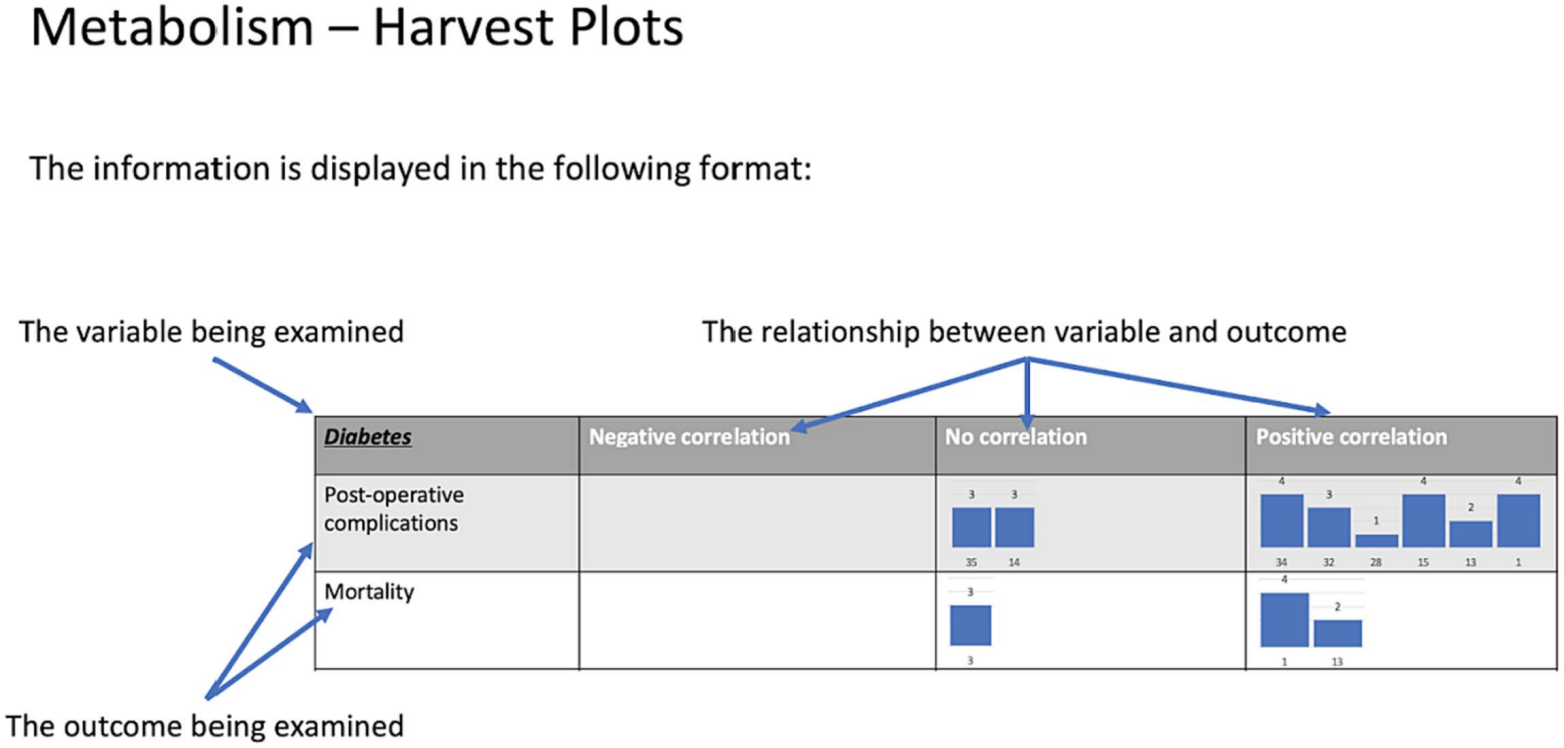

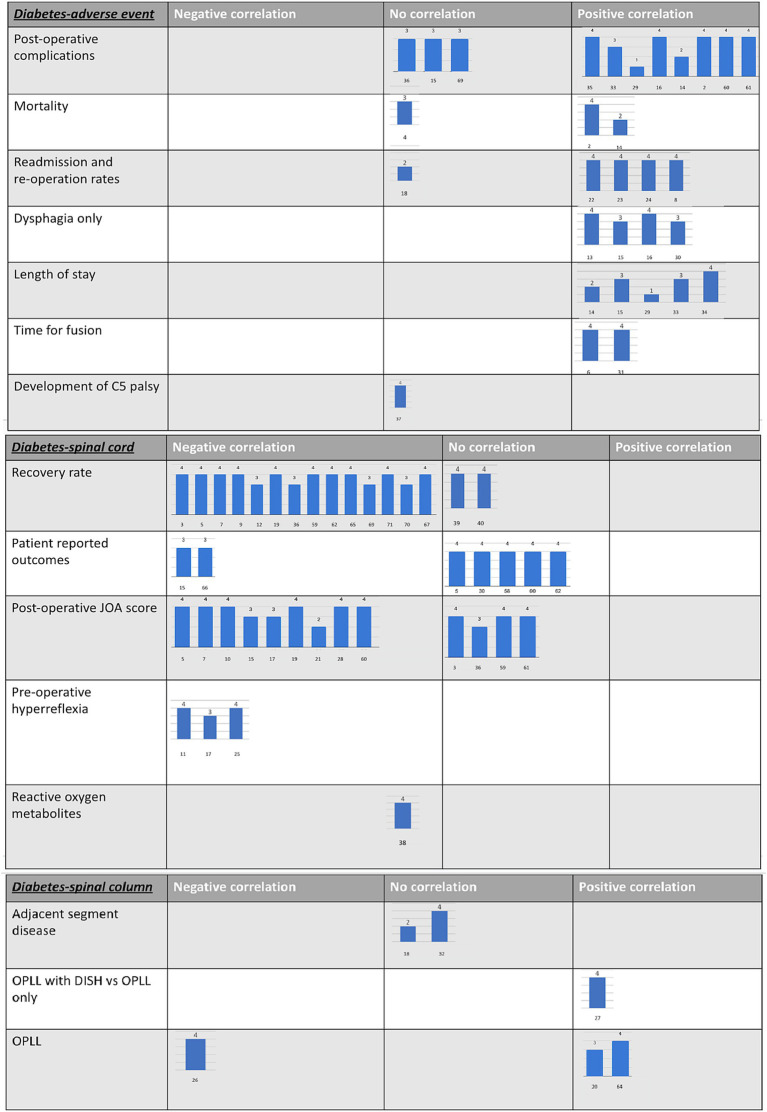

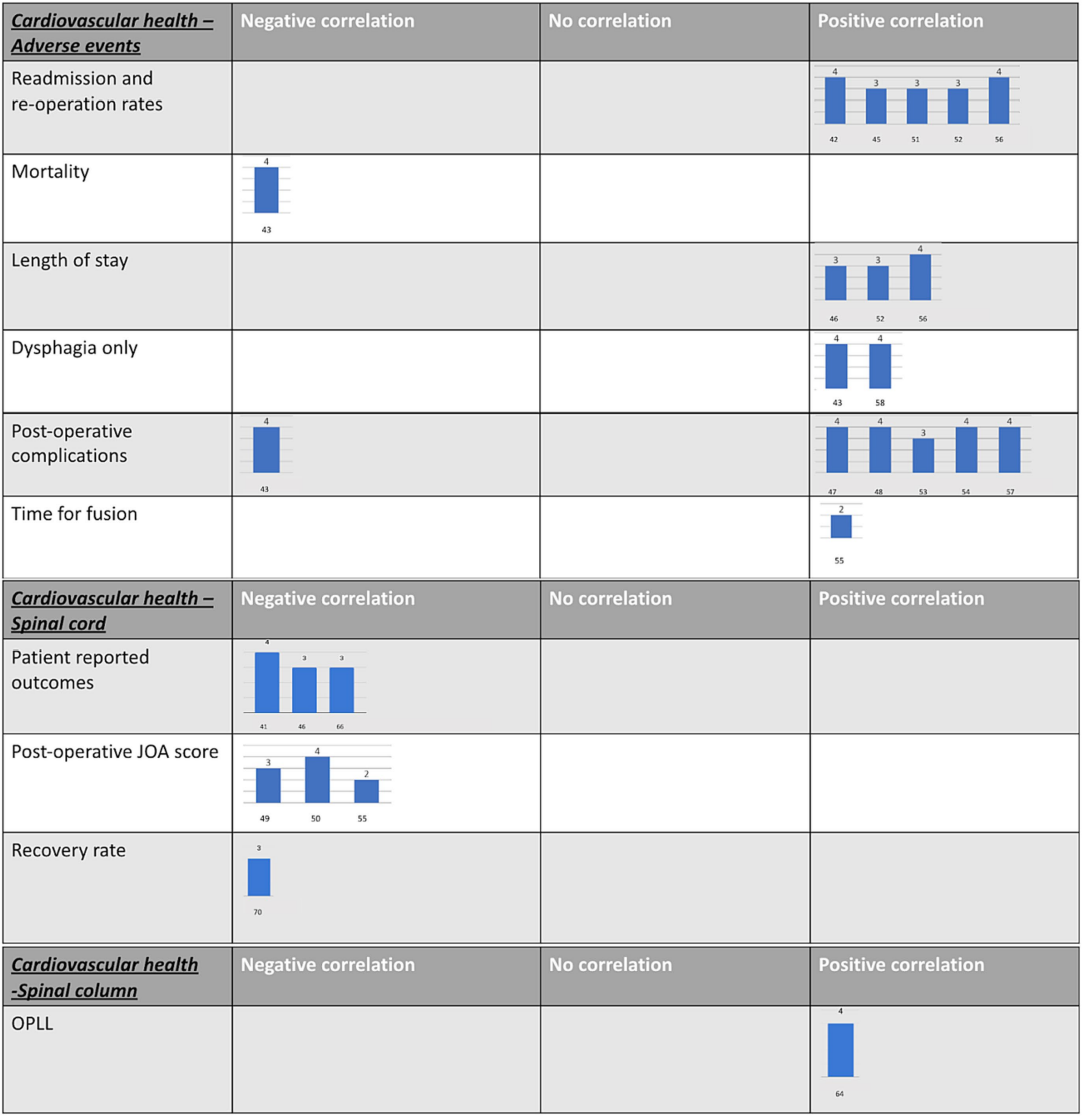
Harvest plots. These were created to provide a visual representation of the GRADE tool results for each paper. **(A)** A guide on how to interpret the harvest plots. **(B)** Diabetes harvest plots. **(C)** Cardiovascular harvest plots.

Confidence in the body of evidence for included studies was assessed using the Grading of Recommendations, Assessment, Development and Evaluations (GRADE) framework (31). A harvest plot was created to provide a visual representation of the GRADE tool results for each paper (Figure 1).

## Results

**Figure 2:**
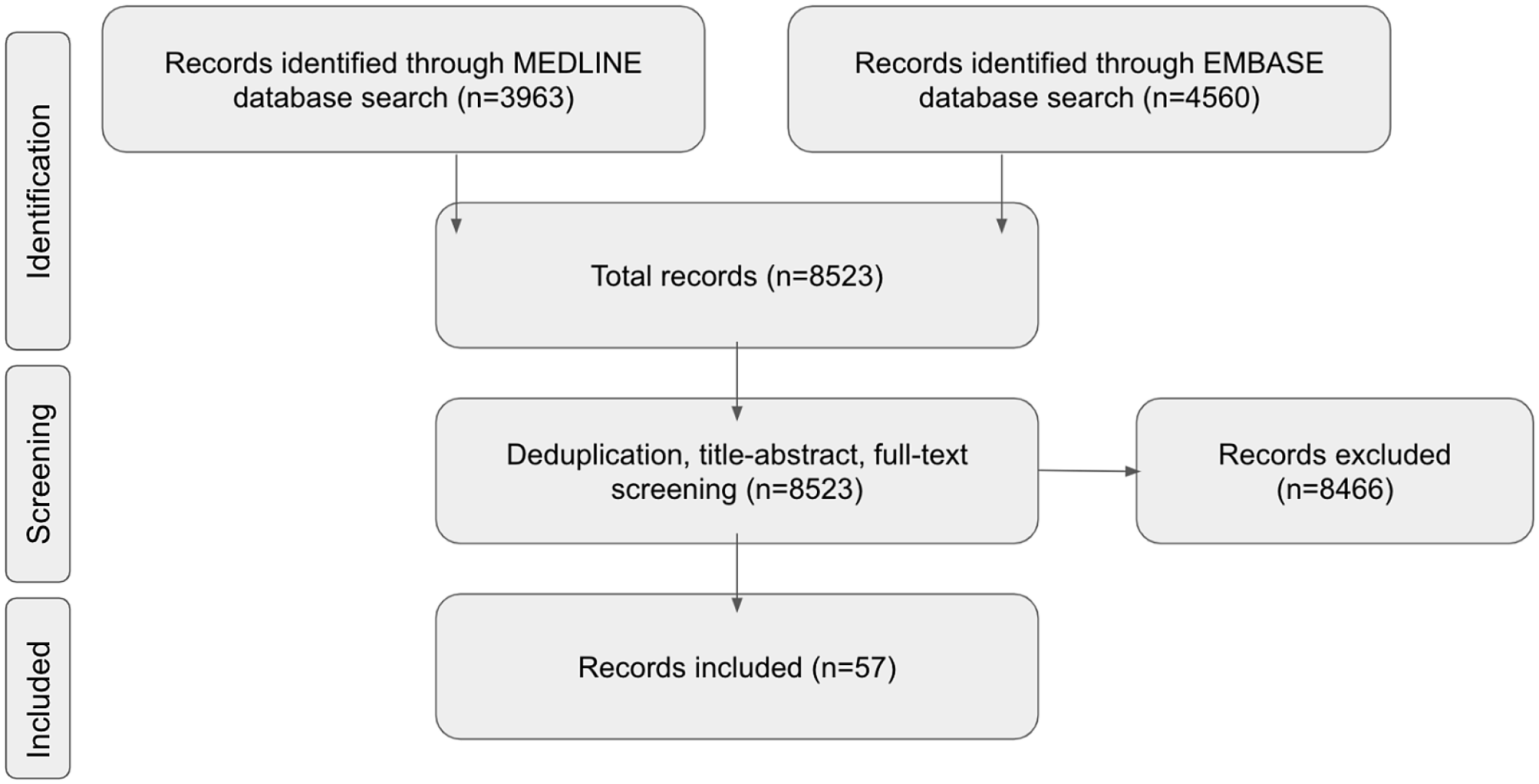
PRISMA flow diagram.

A total of 8,523 papers were screened, identifying 57 articles focused on metabolic disease in the context of DCM (Figure 2). Of these, 52/57 papers (91%) assessed the effect of diabetes, and 24/57 (42%) papers assessed cardiovascular health. The majority (50/57, 88%) were observational cohort studies (prospective or retrospective), with cohort sizes ranging from 24 to 202,694. The remaining were cross-sectional studies (7/57, 12%). The majority studied surgical cohorts (55/57, 96%). The confidence in the body of evidence from the included studies using the GRADE framework is outlined in Table 1.

**Table 1:** Summary and certainty of evidence.

Theme (total papers)	Relationship examined	Total papers	Outcome measured (GRADE score)			
			Main adverse events	Other adverse events	Spinal cord biology	Spinal column biology
1. Diabetes (52)	Significant correlation between diabetes and DCM development	44 [32–52] [54-76]	Post-operative complications (⨁⨁⨁⨁ HIGH)	Length of stay (⨁⨁◯◯ LOW)	Recovery rate (⨁⨁⨁◯ MODERATE)	Adjacent segment disease (⨁◯◯◯ VERY LOW)
			Mortality (⨁⨁◯◯ LOW)		Patient reported outcomes (⨁◯◯◯ VERY LOW)	
	No significant correlation between diabetes and DCM development	15 [33, 36, 45, 53, 60, 64, 65, 67, 74, 77–83]	Re-admission/re-operation (⨁⨁◯◯ LOW)	Development of C5 palsy (⨁◯◯◯ VERY LOW)	Post-operative Japanese Orthopaedic Association score (⨁⨁◯◯ LOW)	OPLL with DISH vs. OPLL only (⨁⨁◯◯ LOW)
			Dysphagia only (⨁⨁◯◯ LOW)		Pre-operative hyper-reflexia (⨁⨁◯◯ LOW)	OPLL (⨁⨁◯◯ LOW)
			Time for fusion (⨁⨁◯◯ LOW)		Reactive oxygen metabolites (⨁◯◯◯ VERY LOW)	
2. Cardiovascular disease	Significant correlation between CVD and DCM development	21 [34, 37, 43, 45, 46, 48, 50, 51, 53, 58, 59, 60, 62, 63, 69, 71, 75, 84–87]	Dysphagia only (⨁◯◯◯ VERY LOW)	Length of stay (⨁◯◯◯ VERY LOW)	Patient reported outcomes (⨁◯◯◯ VERY LOW)	OPLL (⨁⨁◯◯ LOW)
			Post-operative complications (⨁⨁⨁◯ MODERATE)			
			Time for fusion (⨁⨁◯◯ LOW)		Post-operative Japanese Orthopaedic Association score (⨁◯◯◯ VERY LOW)	
			Re-admission/re-operation (⨁◯◯◯ VERY LOW)			
			Mortality (⨁⨁◯◯ LOW)		Recovery rate (⨁⨁◯◯ LOW)	
	No significant correlation between CVD and DCM development	0				

Papers assessing the impact of a particular variable (e.g., weight) on various specific outcomes (e.g., post-operative complications) were included and assessed using the standardised GRADE framework to arrive at a final grade (in brackets) representing the overall quality of studies included for that specific outcome. These outcomes were then grouped into the broader categories of adverse events, spinal cord biology, spinal column biology and other. Definitions: Post-operative complications—the complications measured in papers included under this outcome consist of: concurrent cervical spinal cord compression, superficial site infections, deep site infections (fascial and muscle layers), organ or space site infections (any location apart from the operational incision), reintubation, anterior haematoma evacuation, spinal epidural haematoma, pseudoarthrosis, hardware failure, screw malposition, C5 radiculopathy, axial pain, new intractable neck pain, adjacent segment degeneration, instability, dural tear, neurological deterioration, progression of myelopathy, cortical blindness, non-union, graft dislodgment/migration, graft site pain, postoperative kyphosis, cardiopulmonary event, stroke, deep vein thrombosis, venous thromboembolism, renal complication, peripheral nerve injury, blood transfusions required, urinary tract infections, wound complications, aspiration and acute respiratory distress syndrome. Patient reported outcomes—papers included under this outcome used the following patient questionnaires and scoring systems: the neck disability index (NDI—assesses how neck pain is impacting daily life), short form 36 health survey (SF-36—measures the impact of clinical interventions on daily life using both physical and mental components) and visual analogue scale scores for neck and arm pain.

### Diabetes

A total of 52 papers studied DCM in the context of diabetes (32–83). In total, 44 papers reported that diabetes was associated with poorer surgical outcomes (32–52, 54–76), whilst no association was reported in differing outcome measures across 15 papers (33, 36, 45, 53, 60, 64, 65, 67, 74, 77–83) (Table 1; Figure 1B).

#### Prevalence of diabetes and other comorbidities in DCM patients

In DCM patients, diabetes was reported to be associated with several other comorbidities, all of which appear more significant with age (45, 49, 50, 58). In addition, a cohort study of 9,071 patients comparing myelopathy and radiculopathy patients found that on average myelopathy patients were older, more likely to be male and had higher rates of diabetes (38).

#### Spinal column disease and diabetes

A retrospective study of 49 patients with ossification of the posterior longitudinal ligament (OPLL), of which eight also had combined diffuse idiopathic skeletal hyperostosis (DISH), reported that patients with a combination of OPLL and DISH were significantly more likely to have diabetes compared to those with only OPLL (56). However, one study of 23 patients with OPLL reported that OPLL occurrence was significantly higher in non-diabetics than in diabetics (55). Furthermore, a study of 39 patients that developed adjacent segment disease (ASD) after anterior cervical discectomy and fusion (ACDF) reported that diabetes was not a significant predictor for the development of ASD after ACDF (81).

#### Spinal cord disease and diabetes

A single-centre Singaporean cohort study of 58 patients (29 diabetic vs. 29 non-diabetic) identified that DCM patients were less satisfied following single-level anterior cervical discectomy and fusion (ACDF) if they were diabetic, with surgery more likely to not meet patient expectations, although this difference was not statistically significant (36). A cohort study of 87 patients that had undergone cervical laminoplasty reported that the Japanese Orthopaedic Association (JOA) score improved significantly in both diabetics and non-diabetics; however, the mean post-operative JOA score and mean recovery rate were significantly higher in non-diabetic patients (35). The same study reported that older diabetic patients with a longer history of symptomatic DCM were also more likely to have a poorer recovery rate (35).

A single-centre cohort study of 78 DCM patients undergoing expansive laminoplasty showed that diabetics had poorer recovery of their motor and sensory function in their lower extremities, with a significant negative correlation between pre-operative glycated haemoglobin (HbA1c) and the 6-month recovery rate (33). Two studies reported that post-operative persistence of gait disturbance, hand numbness and bladder dysfunction occurred significantly more in diabetics undergoing cervical laminoplasty (37, 47). One cohort study, consisting of a total of 505 DCM patients, reported that recovery of lower extremity motor and upper extremity sensory function were significantly lower in the diabetic patients (37). The study also reported that the diabetic group had lower pre-and post-operative JOA scores and lower recovery rate of JOA scores (37). However, they also reported that the mean recovery rates of upper extremity motor function after laminoplasty was not significantly different between diabetics and non-diabetic groups (37).

A prospective cohort study of 61 patients showed that JOA scores improved significantly in both diabetic and non-diabetic groups after surgery, with no significant inter-group differences identified (79). However, patients with better control of HbA1c after 12 months had significantly better scores on the Japanese Orthopaedic Association Cervical Myelopathy Evaluation Questionnaire (JOACMEQ), a questionnaire completed by patients to assess the severity of their cervical myelopathy and quality of life. In addition, there were no significant differences in the upper or lower limb function between the two groups (79).

Furthermore, three studies assessed the differences in the reflexes preoperatively between diabetic and non-diabetic patients (41, 47, 54). Diabetics were reported to have a lower prevalence of hyperreflexia and a higher incidence of hyporeflexia (47, 54). Furthermore, a retrospective comparative study of 111 patients that had undergone laminoplasty for DCM reported that Hoffmann’s and Trömner’s reflexes were significantly less common in severely diabetic DCM patients compared to mild diabetics (41). However, the same study also reported no significant difference in the positivity of Babinski’s reflex or the 10-s test (a test of frequency of finger grip and release in 10 s) between those with severe diabetes, mild diabetes and no diabetes (41). Another retrospective study of 438 DCM patients, of which 79 were diabetic, reported no significant difference in Hoffman’s sign between diabetic and non-diabetic patients and found that diabetic patients had a higher incidence of Babinski’s sign (47). Finally, a case–control study of 76 patients reported that diabetic and non-diabetic DCM patients exhibit similar rates of both Hoffmann’s and Babinkski’s sign (54).

#### Surgical adverse events and diabetes

Diabetes has been found to be associated with significantly increased rates of reoperation and surgical complications (38, 44). For example, a single-centre cohort study of 105 patients reported that HbA1c levels greater than or equal to 6.5%, and a duration of diabetes of 10 or more years, were significant risk factors for poor surgical outcome; the same study showed that fasting blood glucose did not affect outcomes (39). In a Canadian survey of 916 surgeons, diabetes was identified as the most important comorbidity affecting surgical fusion outcome, risk of reoperation and readmission in DCM patients (63). In addition, diabetes was reported to significantly increase the risk of perioperative dysphagia and dysphonia in DCM patients undergoing anterior cervical surgery (43, 46, 59). Furthermore, there were significantly poorer fusion outcomes after anterior cervical discectomy and fusion in 29 diabetic DCM patients compared to 29 non-diabetic controls at 2 years postoperatively (36).

A multicentre study of 50,000 patients showed that the presence of uncomplicated or complicated diabetes significantly increased the likelihood of perioperative morbidity in DCM patients undergoing surgery (34). Uncontrolled diabetes was shown to significantly increase the likelihood of mortality, cardiac complications, haematoma, post-operative infection and non-routine discharge in cervical myelopathy patients (44, 64), in addition to unplanned intubation, use of a ventilator for more than 48 h, urinary tract infection, deep vein thrombosis and thrombophlebitis (61).

Type 1 diabetics were more likely to suffer from post-operative neurological, cardiovascular, pulmonary, thromboembolic and renal complications (32, 44). Two studies compared the effects of type 1 and type 2 diabetes on DCM surgical outcomes: a multi-centre cohort study of 1,560 cervical corpectomy patients reported a 4-fold higher mortality rate for type 1 diabetes compared with those with no history of diabetes or diet-controlled diabetes (32). Furthermore, a retrospective cohort study of 37,732 cervical spinal fusion patients showed that those with type 1 diabetes had a higher in-hospital mortality rate and longer average length of stay than type 2 diabetics (44).

However, other studies found differing results: despite diabetic patients having a significantly higher prevalence of comorbidities such as hypertension, hyperlipidaemia and anti-coagulant and anti-platelet use, one cohort study of 500 DCM patients reported no statistically significant difference in the follow-up period, operation time, blood loss, postoperative cervical alignment and range of motion (ROM) between diabetics and non-diabetics (37, 48).

### Cardiovascular health

A total of 24 papers studied the relationship between cardiovascular health and DCM (34, 38, 43, 45, 46, 48, 50, 51, 53, 58–60, 62, 63, 69–72, 75, 84–88) (Table 1; Figure 1C).

#### Spinal cord disease and cardiovascular disease

Cardiovascular disease (CVD) was shown to significantly lower post-surgical patient-reported outcomes, including patient quality of life measured with the Short Form-36 scale amongst 154 DCM patients (84).

#### Surgical adverse events and cardiovascular disease

A survey of 916 surgeons reported a history of angina, coronary artery disease and myocardial infarction as risk factors for surgical complications in DCM patients (63). Coexisting cardiovascular disease (CVD), particularly hypertension, was associated with the greatest risk of all studied comorbidities for post-operative complications in 479 DCM patients undergoing surgery (46), and significantly increased length of hospital stay in 1693 patients undergoing anterior surgeries (62). The five commonest comorbidities that were found to be associated with complications during post-operative rehabilitation included peripheral vascular disease, ischaemic heart disease, stroke, hypertension and diabetes mellitus (58).

Cardiovascular disease was also shown to be a risk factor for the development of perioperative dysphagia in 470 DCM patients undergoing anterior cervical surgery (43). A retrospective cohort study of 3,401 patients following posterior cervical fusion reported pulmonary embolism as a significant predictor for hospital readmission within 30 days after surgery (85). Moreover, in a retrospective national database analysis of 202,694 patients, congestive heart failure and pulmonary circulation disorders were shown to significantly increase the risk of pulmonary aspiration during cervical spine surgery (86).

A multi-centre cohort study of 3,057 showed that patients undergoing posterior surgeries or combined spinal procedures were more likely to be hypertensive than those undergoing anterior approaches (62). Additionally, a case control study of 32 out of 8,250 patients who developed postoperative spinal epidural haematoma (SEH) following spinal decompression reported that, although SEH patients had a higher prevalence of hypertension and coagulopathy than the control group, these differences were not statistically significant (87). However, a multi-centre study of over 54,000 surgical DCM patients reported that hypertension significantly decreased the risk of mortality (34). Currently, expert opinions on anterior vs. posterior surgical approaches tend to be based on cervical sagittal alignment parameters (89). However, the above cardiovascular factors may also have a role in decision making regarding surgical management.

### Risk of bias

**Figure 3:**
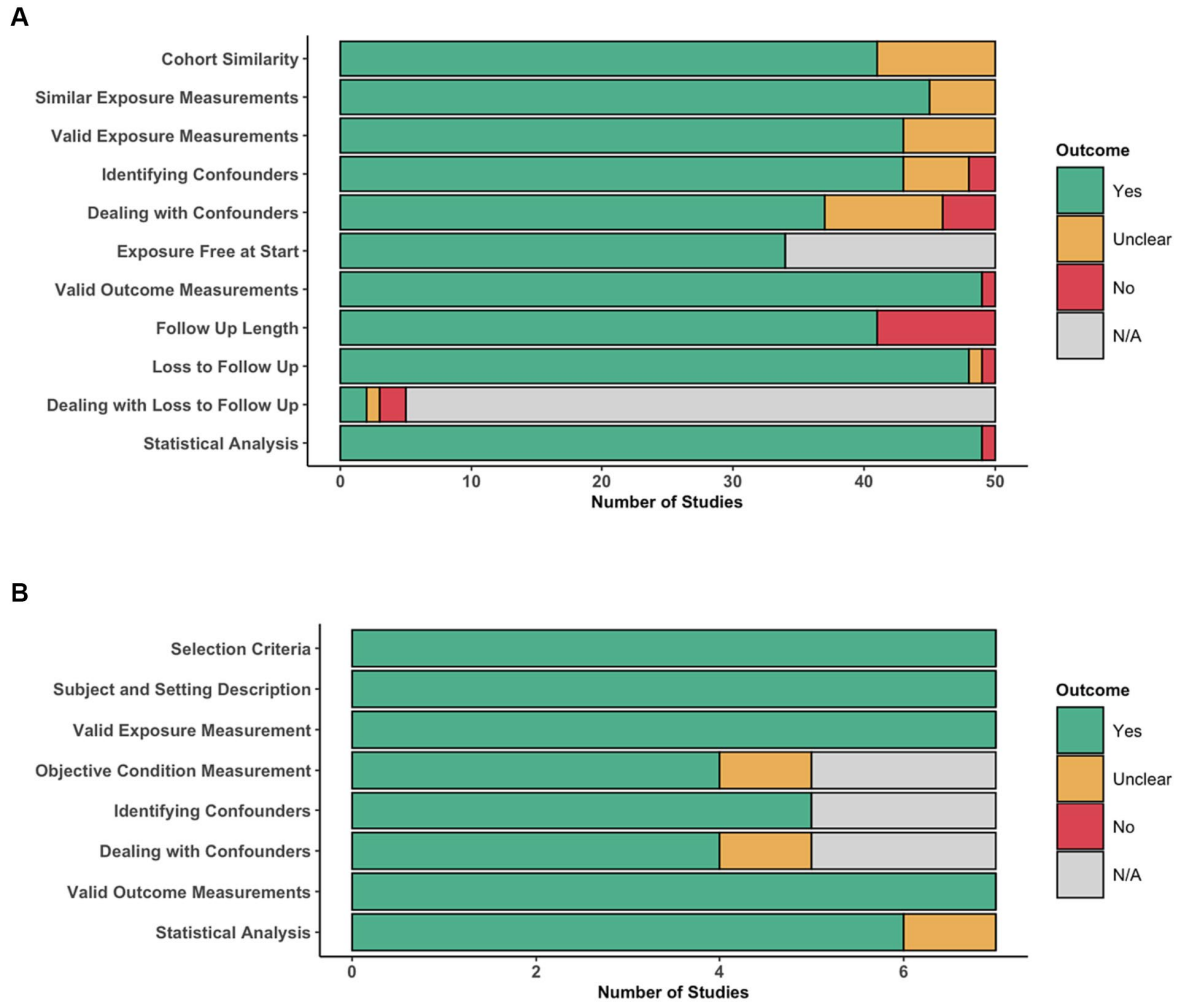
Risk of bias assessment. Distribution of per-item scores for cohort studies, indicating for each item, the number of articles scoring ‘Yes’, ‘Unclear’, ‘No’, or ‘N/A’. Distribution of per-item scores for cross-sectional studies, indicating for each item, the number of articles scoring ‘Yes’, ‘Unclear’, ‘No’, or ‘N/A’.

The distribution of assessment of individual items of the JBI critical appraisal tool for cohort studies is depicted in Figure 3A. The similarity of the cohorts used in 10 of the included studies were deemed unclear (32, 35, 36, 49, 52, 56, 58, 60, 61, 86), mainly due to a lack of clear selection criteria. Measurement of exposures was mostly adequate, except in five studies (36, 47, 49, 52, 66), where the reporting of how exposures were measured were not deemed to be detailed enough. Similarly, seven studies did not include sufficient detail to be able to confidently conclude that exposures were measured in a valid and reliable manner (37, 47, 49, 52, 53, 55, 66). Identification of confounding factors (e.g., sex, age and duration of symptoms) was mostly adequate, except in six studies that appeared to miss key confounders (47, 52, 56, 58, 60, 61), most commonly due to lack of complete exclusion criteria, and two studies that appeared to be missing most confounders (32, 49). Strategies to deal with confounders was performed to a lower quality, with 13 studies deemed unclear (38, 39, 50, 52, 53, 55, 58, 60, 61, 66, 73, 77, 79), and four studies deemed inadequate (32, 47, 48, 56), most commonly due to a lack of a multivariate regression analysis. The cohorts were deemed either free of the outcome at the start of all studies or this criteria was not applicable, if for example the outcome was improvement in neurological status. The outcomes were measured in a valid and reliable manner in most studies, except one (49), where the methods section was sparse. The follow-up time was mostly adequate in length and reporting, except in nine studies (34, 44, 47, 49, 55, 58, 61, 73, 86), often due to incomplete reporting. Follow-up was mostly adequate, often owing to the retrospective nature of a significant portion of included studies, or if there was substantial loss to follow-up, this was usually reasonably explored, except in two studies (46, 66). As a result, most studies did not need to provide information on how incomplete follow-up would be handled. All, except for one study (49), appeared to use appropriate statistical analysis.

The distribution of assessment of individual items of the JBI critical appraisal tool for cross-sectional studies is depicted in Figure 3B. On the whole, cross-sectional studies were deemed to have low risk of bias, addressing all of the criteria adequately, except for one study (54), where their strategies to deal with confounding factors and use of statistical analysis were unclear.

## Discussion

The objective of this systematic review was to synthesise the current evidence on metabolic dysfunction in DCM. Our synthesis shows that metabolic factors appear to have an impact on outcomes in DCM. This association is strongest with respect to surgical adverse events, but also exists for spinal cord recovery following surgery. However, studies have not sufficiently evaluated the significance of metabolic factors with respect to the onset of DCM, although they do appear to affect initial clinical assessment. Furthermore, it remains uncertain whether these factors are modifiable. These remain important knowledge gaps and areas for future targeted research (10).

Spinal cord ischaemia is a common feature in pre-clinical models and autopsy specimens of DCM and has been proposed as a final common pathway of spinal cord injury resulting from critical cord compression (11). For example, Ellingson et al. (90) used MRI to evaluate spinal cord perfusion and demonstrated that neurological function using the modified JOA (mJOA) was inversely correlated with oxygen extraction. Although inter-rater reliability of total mJOA and its subscores are useful, mJOA should be interpreted carefully, particularly when near the threshold between severity categories, or when a patient is reassessed for deterioration (91). Moreover, the relationship is likely to be bidirectional, with systemic factors influencing the spinal cord, but also the spinal cord influencing systemic cardiovascular disease. For example, autonomic dysfunction can arise with spinal cord damage including DCM (92–94), and a recent Taiwanese population study identified DCM as an independent risk factor for the occurrence of acute coronary syndromes (95).

Implications for the systemic circulation, and therefore, metabolic disease would seem logical. The aggregated clinical evidence here aligns with this, with studies demonstrating poorer pre-and post-operative neurological function (41, 47, 54). Whilst there is need for further investigation due to existing studies being few in number and generally low in quality, what is clear from the data presented here, is that this line of enquiry will be challenging, due to the significant interaction of these factors. Moreover, the balance of evidence strongly associates metabolic disease with surgical complications, including cardiovascular disease (38, 43, 46, 53, 62, 87) and diabetes (33, 37–39, 44, 46, 47, 56). Whilst this fits with wider surgical experience (96), it will confound the use of post-operative recovery as a surrogate measure to investigate this relationship.

Furthermore, these individual diseases interact and have their own levels of within factor significance. For example, diabetic DCM patients often present with several other confounding conditions, which could worsen their post-operative function (82, 97); these include hypertension, hyperlipidaemia and a procoagulant state (48, 98). Other studies have shown that diabetes and smoking are perhaps the most important risk factors for development of dysphagia (59), but these two factors were shown to coexist with CVD (98). Furthermore, type 1 diabetics were shown to have greater post-operative neurological, cardiovascular, pulmonary, thromboembolic and renal complications than type 2 diabetics (32, 44), and duration of diabetes for over a decade was a significant predictor for poorer surgical outcomes (39). These diseases are also influenced by many unmeasured variables, such as diet and lifestyle (99). So, whilst we might hypothesise that autonomic neuropathy, prevalent and very often subclinical in diabetes, could be a major contributor for these problems, confirming this will be challenging (100).

This complexity is well demonstrated by Badhiwala et al. (84), who used a principal component analysis to explore different clinical phenotypes based on comorbidities and recovery profiles within the AO Spine datasets; they demonstrated that cardiovascular, renal and gastric comorbidities were statistically significant patient characteristics, with ‘eigenvalues >1’, and thus may significantly impact post-surgical outcome in DCM patients. However, once again, the complexity is important to appreciate when making such conclusions.

Whilst the specific impact of metabolic disease on the acquisition of DCM remains theoretical, the burden of cardiovascular disease and implications for surgery, in a condition predominantly treated with surgery, indicate a need to focus research on this question. Across surgery, these factors are considered broadly modifiable or at least suitable for optimisation, either pre-or peri-operatively (101, 102), increasingly termed prehabilitation (103). This is therefore relevant even in context of time constraints, where DCM surgery can be time critical (104, 105).

One additional finding of note was the implications for diabetes on examination findings, albeit inconsistent, in particular the presence or absence pathological reflexes differing between studies (41, 47, 54). Given its restriction to reflexes, these observations may well be driven by a subclinical and co-existent peripheral neuropathy, which is extremely prevalent (~30–50%) amongst diabetics (106). However peripheral diabetic neuropathy is also recognised to manifest other neurological implications, including gait and motor dysfunction (107). This may have implications for diagnosis, where expected examination findings may be mute, or outcome assessments, where measures may be confounded (108, 109). Supporting the former is an AO Spine RECODE DCM research priority^1^ owing to the significant under, mis-and delayed diagnosis increasing disability and dependence (105, 110–114).

### Limitations

The findings of this study are limited to the existing evidence base, which is low in quality and selective in its focus, largely orientated to surgery, in particular anterior surgery. However overall, the balance of current evidence supports metabolism being important in DCM. What is missing is any proof of causation and elucidation of the fundamental mechanisms. This is especially pertinent since many aforementioned metabolic factors are likely to interact. Contradiction between studies exists for several topics and control of confounding factors has generally been poor, making generalisability of correlations limited. Studies mainly included DCM patients without diabetes or CVD as controls, however, it would also be useful to compare metabolically impaired DCM patients against non-DCM groups, highlighting a knowledge gap that requires further investigation. Given the role of metabolism in DCM may be like that in many other conditions, an initial broad approach focusing on what is known about metabolism in better-researched neurological conditions may also be appropriate. This is likely to require a large, and high-quality dataset, capturing all relevant determinants, including patient demographics such as age, ethnicity, and weight, but also diet and lifestyle.

## Conclusion

Metabolic disease increases the risks of adverse events in patients undergoing surgical treatment for DCM (GRADE moderate strength evidence). Given the recognised potential for this to be beneficially modified in other surgical fields, alongside the putative and low-quality clinical evidence indicating a potential significant relationship between metabolic disease and spinal cord function and recovery, this research area merits further investigation. The differing examination findings amongst DCM patients with diabetes is also of relevance to those investigating strategies for earlier diagnosis of DCM. Future directions of DCM management would certainly rely on studies meant to address the knowledge gap on the role of metabolic factors in the decision-making process for DCM (115).

## Data availability statement

The original contributions presented in the study are included in the article/Supplementary material, further inquiries can be directed to the corresponding author.

## Author contributions

CP: Writing – original draft, Conceptualization, Data curation, Formal analysis, Investigation, Methodology, Validation. AS: Data curation, Writing – review & editing, Formal analysis, Investigation, Methodology. AR: Data curation, Formal analysis, Investigation, Methodology, Validation, Visualization, Writing – review & editing. FB: Data curation, Writing – review & editing. TR: Data curation, Writing – review & editing. SA: Data curation, Writing – review & editing. MA: Data curation, Writing – review & editing. AB: Data curation, Writing – review & editing. AN: Supervision, Writing – review & editing. MK: Supervision, Writing – review & editing. BD: Conceptualization, Methodology, Supervision, Validation, Writing – review & editing. OM: Conceptualization, Methodology, Supervision, Validation, Writing – review & editing.

## Footnotes

^1^ aospine.org/recode
