# Supplementary material for: The significance of metabolic disease in degenerative cervical myelopathy: a systematic review
Source: Front Neurol. 2024 Feb 5;15:1301003. doi: 10.3389/fneur.2024.1301003 (PMC10876002; doi:10.3389/fneur.2024.1301003)

**Supplementary Data 1 – PRISMA Checklist**

| **Section and Topic** | **Item #** | **Checklist item** |
| --- | --- | --- |
| **TITLE** | | |
| Title | 1 | Identify the report as a systematic review. |
| **ABSTRACT** | | |
| Abstract | 2 | See the PRISMA 2020 for Abstracts checklist. |
| **INTRODUCTION** | | |
| Rationale | 3 | Describe the rationale for the review in the context of existing knowledge. |
| Objectives | 4 | Provide an explicit statement of the objective(s) or question(s) the review addresses. |
| **METHODS** | | |
| Eligibility criteria | 5 | Specify the inclusion and exclusion criteria for the review and how studies were grouped for the syntheses. |
| Information sources | 6 | Specify all databases, registers, websites, organisations, reference lists and other sources searched or consulted to identify studies. Specify the date when each source was last searched or consulted. |
| Search strategy | 7 | Present the full search strategies for all databases, registers and websites, including any filters and limits used. |
| Selection process | 8 | Specify the methods used to decide whether a study met the inclusion criteria of the review, including how many reviewers screened each record and each report retrieved, whether they worked independently, and if applicable, details of automation tools used in the process. |
| Data collection process | 9 | Specify the methods used to collect data from reports, including how many reviewers collected data from each report, whether they worked independently, any processes for obtaining or confirming data from study investigators, and if applicable, details of automation tools used in the process. |
| Data items | 10a | List and define all outcomes for which data were sought. Specify whether all results that were compatible with each outcome domain in each study were sought (e.g. for all measures, time points, analyses), and if not, the methods used to decide which results to collect. |
|  | 10b | List and define all other variables for which data were sought (e.g. participant and intervention characteristics, funding sources). Describe any assumptions made about any missing or unclear information. |
| Study risk of bias assessment | 11 | Specify the methods used to assess risk of bias in the included studies, including details of the tool(s) used, how many reviewers assessed each study and whether they worked independently, and if applicable, details of automation tools used in the process. |
| Effect measures | 12 | Specify for each outcome the effect measure(s) (e.g. risk ratio, mean difference) used in the synthesis or presentation of results. |
| Synthesis methods | 13a | Describe the processes used to decide which studies were eligible for each synthesis (e.g. tabulating the study intervention characteristics and comparing against the planned groups for each synthesis (item #5)). |
|  | 13b | Describe any methods required to prepare the data for presentation or synthesis, such as handling of missing summary statistics, or data conversions. |
|  | 13c | Describe any methods used to tabulate or visually display results of individual studies and syntheses. |
|  | 13d | Describe any methods used to synthesize results and provide a rationale for the choice(s). If meta-analysis was performed, describe the model(s), method(s) to identify the presence and extent of statistical heterogeneity, and software package(s) used. |
|  | 13e | Describe any methods used to explore possible causes of heterogeneity among study results (e.g. subgroup analysis, meta-regression). |
|  | 13f | Describe any sensitivity analyses conducted to assess robustness of the synthesized results. |
| Reporting bias assessment | 14 | Describe any methods used to assess risk of bias due to missing results in a synthesis (arising from reporting biases). |
| Certainty assessment | 15 | Describe any methods used to assess certainty (or confidence) in the body of evidence for an outcome. |
| **RESULTS** | | |
| Study selection | 16a | Describe the results of the search and selection process, from the number of records identified in the search to the number of studies included in the review, ideally using a flow diagram. |
|  | 16b | Cite studies that might appear to meet the inclusion criteria, but which were excluded, and explain why they were excluded. |
| Study characteristics | 17 | Cite each included study and present its characteristics. |
| Risk of bias in studies | 18 | Present assessments of risk of bias for each included study. |
| Results of individual studies | 19 | For all outcomes, present, for each study: (a) summary statistics for each group (where appropriate) and (b) an effect estimate and its precision (e.g. confidence/credible interval), ideally using structured tables or plots. |
| Results of syntheses | 20a | For each synthesis, briefly summarise the characteristics and risk of bias among contributing studies. |
|  | 20b | Present results of all statistical syntheses conducted. If meta-analysis was done, present for each the summary estimate and its precision (e.g. confidence/credible interval) and measures of statistical heterogeneity. If comparing groups, describe the direction of the effect. |
|  | 20c | Present results of all investigations of possible causes of heterogeneity among study results. |
|  | 20d | Present results of all sensitivity analyses conducted to assess the robustness of the synthesized results. |
| Reporting biases | 21 | Present assessments of risk of bias due to missing results (arising from reporting biases) for each synthesis assessed. |
| Certainty of evidence | 22 | Present assessments of certainty (or confidence) in the body of evidence for each outcome assessed. |
| **DISCUSSION** | | |
| Discussion | 23a | Provide a general interpretation of the results in the context of other evidence. |
|  | 23b | Discuss any limitations of the evidence included in the review. |
|  | 23c | Discuss any limitations of the review processes used. |
|  | 23d | Discuss implications of the results for practice, policy, and future research. |
| **OTHER INFORMATION** | | |
| Registration and protocol | 24a | Provide registration information for the review, including register name and registration number, or state that the review was not registered. |
|  | 24b | Indicate where the review protocol can be accessed, or state that a protocol was not prepared. |
|  | 24c | Describe and explain any amendments to information provided at registration or in the protocol. |
| Support | 25 | Describe sources of financial or non-financial support for the review, and the role of the funders or sponsors in the review. |
| Competing interests | 26 | Declare any competing interests of review authors. |
| Availability of data, code and other materials | 27 | Report which of the following are publicly available and where they can be found: template data collection forms; data extracted from included studies; data used for all analyses; analytic code; any other materials used in the review. |

**Supplementary Data 2 – Search Strategy**

POPULATION:

| # | MEDLINE (from Davies, 2018) | Hits | EMBASE 1980-2020 week 4 | Hits |
| --- | --- | --- | --- | --- |
| 1 | exp Cervical Vertebrae/ or exp Cervical Cord/ or cervical.mp. or (phrenic nucleus or accessory nucleus).mp. or (("Japanese Orthop?edic Association" adj2 score*) or (joa adj2 score*)).mp. | 257565 | exp *cervical spinal cord/ or cervical.ti,ab. or exp *cervical spine/ or exp *cervical vertebra/ or (phrenic nucleus or accessory nucleus).ti,ab. or exp *Japanese Orthopaedic Association score/ or Japanese Orthop?edic Association.ti,ab. or ("Japanese Orthop?edic Association" adj2 scor*).ti,ab. or (joa adj2 scor*).ti,ab. | 249509 |
| 2 | myelopath*.mp. or exp Spinal Cord Diseases/ or (spinal cord adj3 (diseas* or disorder*)).mp. or myeloradiculopath*.mp. or spondylomyelopath*.mp. or spondylomyeloradiculopath*.mp. or (Spinal Cord adj3 Compress*).mp. or exp Spinal Cord Compression/ | 139719 | myelopath*.ti,ab. or exp cervical myelopathy/ or exp *cervical spondylotic myelopathy/ or spondylotic cervical myelopathy.mp. or exp *spinal cord disease/ or "cervical spinal cord injury".ti,ab. or exp *myelography/ or exp *myeloradiculopathy/ or myeloradiculopath*.ti,ab. or exp *cervical spondylosis/ or (spinal cord adj3 (diseas* or disorder*)).ti,ab. or spondylomyelopath*.ti,ab. or (Spinal Cord adj3 Compress*).ti,ab. or exp *spinal cord compression/ | 151542 |
| 3 | 1 and 2 | 19050 | 1 and 2 | 20060 |
| 4 | exp "Ossification of Posterior Longitudinal Ligament"/ | 934 | "ossification of posterior longitudinal ligament".ti,ab. or exp *ligament calcinosis/ or (exp *posterior longitudinal ligament/ and (exp *ossification/ or ossifi*.ti,ab.)) | 1397 |
| 5 | 3 or 4 | 19518 | 3 or 4 | 20898 |
| 6 | exp Atlanto-Occipital Joint/ or exp Arteriovenous Fistula/ or exp Radiotherapy/ or exp Vitamin B 12/ or exp Radiation/ or exp Radiation Injuries/ or exp Re-Irradiation/ or exp Craniospinal Irradiation/ or exp Whole-Body Irradiation/ or exp Motor Neuron Disease/ or exp Amyotrophic Lateral Sclerosis/ or exp Neoplasm Metastasis/ or exp Hemangioma/ or exp neoplasm/ or exp metastasis/ or exp Nervous System Malformations/ or exp "autoimmune diseases of the nervous system"/ or exp "congenital, hereditary, and neonatal diseases and abnormalities"/ or exp virus diseases/ | 5670902 | exp atlantooccipital joint/ or exp arteriovenous fistula/ or exp radiotherapy/ or exp cyanocobalamin/ or exp radiation injury repair/ or exp radiation injury/ or exp *radiation/ or exp re-irradiation/ or exp irradiation/ or exp craniospinal irradiation/ or exp whole body radiation/ or exp *motor neuron disease/ or exp *amyotrophic lateral sclerosis/ or neoplasm metastasis.mp. or exp metastasis/ or exp *neoplasm/ or exp malignant neoplasm/ or exp radiation induced neoplasm/ or exp myeloproliferative neoplasm/ or exp vertebra hemangioma/ or exp hemangioma/ or exp nervous system malformation/ or autoimmune diseases of the nervous system.mp. or autoimmune nervous system.mp. or (congenital, hereditary, and neonatal diseases and abnormalities).mp. or "congenital disorder".mp. or exp genetic disorder/ or "newborn disease".mp. or exp virus infection/ | 5801757 |
| 7 | 5 not 6 | 14368 | 5 not 6 | 16462 |

AND

INTERVENTION:

| # | MEDLINE | Hits | EMBASE | Hits |
| --- | --- | --- | --- | --- |
| 1 | Exp food/ or food.mp or exp eating/ or exp carbohydrates/ or carbohydrate.mp or exp meat/ or exp meat products/ or exp meat proteins/ or exp red meat/ or meat.mp or exp vegans/ or vegan.mp or exp vegetarians/ or vegetarian.mp or exp diet/ or diet.mp or exp diet, carbohydrate-restricted/ or exp diet, carbohydrate loading/ or exp diet, fat-restricted/ or exp diet, gluten-free/ or exp diet, high fat/ or exp diet,, high protein/ or exp diet, ketogenic/ or exp diet, Mediterranean/ or exp diet, protein-restricted/ or exp diet, vegan/ or exp diet, vegetarian/ or exp diet, Western/ or exp diet, diabetic/ or exp diet, paleolithic/ or exp fish proteins/ or exp fish proteins, dietary/ or exp egg proteins/ or exp dietary proteins/ or exp proteins/ or exp pea proteins/ or exp whey proteins/ or exp exp healthy diet/ or exp diet, macrobiotic/ or exp dietary supplements/ or supplements.mp or exp enteral nutrition/ or feeding.mp or exp water/ or water.mp or exp body water/ or exp water deprivation/ or nutrition*.mp or exp enteral nutrition/ or exp parenteral nutrition/ or exp malnutrition/ or malnutrition.mp or exp vitamins/ or exp vitamin D/ or exp vitamin D deficiency/ or exp vitamin E/ or exp ascorbic acid/ or exp vitamin B 12/ or exp vitamin B 12 deficiency/ or exp vitamin B complex/ or vitamin.mp or cobalamin C.mp or cyanocobalamin.mp or an?emi*.mp or exp anemia/ or exp anemia, iron-deficiency/ | 9419039 | exp *food/ or food.ti,ab or exp *food intake/ or exp *carbohydrate/ or carbohydrate.ti,ab or exp *meat/ or exp *meat protein/ or exp *red meat/ or exp *white meat/ or exp *vegan/ or exp *vegetarian/ or vegan.ti,ab or meat.ti,ab or vegetarian.ti,ab or exp *diet/ or exp *artificial diet/ or exp *diabetic diet/ or exp *diet induced obesity/ or exp *diet restriction/ or exp *diet supplementation/ or exp *fiber free diet/ or exp *fruitarian diet/ or exp *gluten free diet/ or exp *gluten free casein free diet/ or exp *healthy diet/ or exp *high calorie diet/ or exp *high fiber diet/ or exp *high glycemic index diet/ or exp *ketogenic diet/ or exp *lactose free diet/ or exp *lipid diet/ or exp *liquid diet or exp *low glycemic index diet/ or exp *macrobiotic diet/ or exp *Mediterranean diet/ or exp *obesogenic diet or exp *paleolithic diet/ or exp *pescovegetarian diet/ or exp *protein diet/ or exp *raw food diet/ or exp *renal diet/ or exp *unhealthy diet/ or exp *vegan diet/ or exp *vegetarian diet/ or exp *lactovegetarian diet/ or exp *Western diet/ or diet.ti,ab or supplement.ti,ab or exp *feeding/ or exp *feeding tube/ or exp *intravenous feeding or exp *enteric feeding/ or feeding.ti,ab or exp *water/ or water.ti,ab or nutrition*.ti,ab or exp *nutrition/ or exp *nutrition supplement/ or exp *enteral nutrition/ or exp *parenteral nutrition/ or exp *malnutrition/ or malnutrition.ti,ab or exp *protein calorie malnutrition or exp fetal malnutrition or exp *vitamin/ or exp *vitamin B complex/ or exp *vitamin D/ or exp vitamin intake/ or exp *vitamin deficiency/ or exp *vitamin supplementation/ or vitamin.ti,ab or exp *cyanocobalamin or cobalamin.ti,ab or cyanocobalamin.ti,ab or exp *anemia/ or an?emi*.ti,ab or exp *iron deficiency anemia/ | 2942372 |
| 2 | exp folic acid/ or folic acid.mp or exp zinc/ or zinc.mp or exp copper/ or copper.mp or exp ceruloplasmin/ or ceruloplasmin.mp or hypocupr?emi*.mp or exp iron/ or exp iron overload/ or exp iron, dietary/ or exp iron metabolism disorders/ or exp iron compounds/ or iron.mp | 535590 | exp *folic acid/ or folic acid.ti,ab or exp *zinc/ or exp *zinc deficiency/ or exp *zinc urine level/ or zinc.ti,ab or exp *iron/ or exp *iron deficiency/ or iron.ti,ab or exp *copper/ or exp *copper deficiency/ or exp *copper blood level/ or copper.ti,ab or *exp *ceruloplasmin/ or exp *ceruloplasmin blood level/ or hypocupraemia.ti,ab | 408920 |
| 3 | exp weights and measures/ or weight.mp or exp body weight/ or exp body mass index/ or body mass.mp or exp body height/ or exp waist-height ratio/ or height.mp or exp diabetes mellitus, type 2/ or exp overweight/ or exp obesity/ or exp diabetes mellitus, type 1/ or exp diabetes complications/ or diabetes.mp or exp waist circumference/ or circumference.mp or exp appetite/ or exp appetite depressants/ or exp appetite regulation/ or exp appetite stimulants/ or exp *weight loss/ or exp acute disease/ or acute disease.mp or BMI.mp or eat.mp or exp feeding behavior/ or feeding.mp or exp stress, psychological/ or exp stress, physiological/ or stress.mp or exp dietary fats/ or exp dietary fats, unsaturated/ or exp fats/ or exp fats, unsaturated/ or fat.mp or exp muscular atrophy/ or exp cachexia/ or exp muscular dystrophy, Duchenne/ or exp sarcopenia/ or musc* wasting.mp or musc* atrophy.mp or cachexia.mp or sarcopenia.mp or exp edema/ or oedema.mp or edema.mp or exp bariatrics/ or exp obesity,morbid/ or exp metabolic syndrome or metabol*.mp or exp bariatric surgery/ or bariatric.mp or exp hyperglycemia/ or hyperglyc?emi*.mp or exp hypoglycemia/ or hypoglyc?emi*.mp or exp insulin/ or exp insulin, short-acting/ or exp insulin, long-acting/ or insulin-like growth factor I/ or insulin-like growth factor II/ or exp insulin resistance/ or insulin.mp or exp skinfold thickness/ or exp anthropometry/ or exp body composition/ or thickness.mp or skinfold.mp or exp energy metabolism/ or exp energy intake/ or energy.mp | 9128271 | exp *weight/ or exp *body weight/ or exp *birth weight/ or exp *fetal weight/ or exp *ideal body weight/ or exp *gestational weight gain/ or exp *ideal body weight/ or exp *low birth weight/ or exp *weight height ratio/ or exp *cuff weight/ or weight*.ti,ab or exp *height/ or exp *body height/ or exp *waist to height ratio/ or exp *weight height ratio/ or height.ti,ab or exp *abdominal circumference/ or exp *arm circumference/ or exp *chest circumference or exp *hip circumference/ or exp *neck circumference/ or exp *thigh circumference/ or exp * waist circumference/ or exp *head circumference/ or circumference*.ti,ab or exp *appetite/ or exp *appetite disorder/ or exp *appetite stimulant/ or exp *decreased appetite/ or exp *increased appetite/ or exp *sodium appetite/ or appetite.ti,ab or exp *acute disease or acute disease.ti,ab or exp *BMI chart/ or exp *body mass/ or exp *obesity/ or BMI.ti,ab or body mass.ti,ab or obesity.ti,ab or ability to eat.ti,ab or exp *feeding behaviour/ or exp *stress/ or exp *stress hormone/ or stress.ti,ab or exp *fat/ or exp *body fat/ or exp *fat mass/ or exp *fat free mass/ or exp *low fat diet/ or exp *pericardial fat/ or exp *perirenal fat/ or exp *subcutaneous fat/ or exp *subcutaneous fat disorder/ or fat.ti,ab or exp *muscle atrophy/ or muscle wasting.ti,ab or muscle atrophy.ti,ab or exp *edema/ or edema.ti,ab or oedema.ti,ab or exp *functional status/ or functional status.ti,ab or functional capacity.ti,ab or exp *bariatric surgery/ or bariatric.ti,ab or exp *morbid obesity/ or diabetes.ti,ab or exp *diabetes mellitus/ or exp *hyperglycemia/ or hyperglyc?emi*.ti,ab or exp *insulin/ or exp *insulin dependence/ or exp *insulin resistance/ or exp *long acting insulin/ or exp *short acting insulin/ or exp *pig insulin/ or insulin.ti,ab or exp *body distribution/ or exp *insulin sensitivity/ or skinfold thickness.ti,ab or triceps skinfold.ti,ab or exp *energy/ or exp *energy metabolism/ or exp *energy balance/ or exp *energy absorption/ or exp *energy expenditure/ or energy.ti,ab | 4164312 |
| 4 | exp hyponatremia/ or hyponatr?emi*.mp or exp hyperkalemia/ or hyperkal?emi*.mp or exp methylmalonic acid/ or methylmalonic acid.mp or exp minerals/ or exp antioxidants/ or mineral.mp or exp albumins/ or albumin.mp or exp serum albumin/ or exp prealbumin/ or transthyretin.mp or exp transferrin/ or transferrin.mp or exp ferritins/ or ferritin.mp or exp retinol-binding proteins/ or retinol.mp or RBP.mp or exp nitrogen/ or exp nitrogen compounds/ or exp reactive nitrogen species/ or exp nitrogen oxides/ or nitrogen.mp or exp trace elements/ or trace element.mp or exp selenium/ or selenium.mp or exp selenium compounds/ or exp selenium oxides/ or exp iodides/ or iodide.mp or skin sensitivity.mp or exp C-reactive protein/ or c-reactive protein.mp or CRP.mp or exp inflammation/ or exp inflammation mediators/ or exp neurogenic inflammation/ or inflammat*.mp or exp orosomucoid/ or orosomucoid.mp or alpha-1-acid glycoprotein.mp or exp lymphocyte count/ or exp leukocyte count/ or exp lymphocytes/ or lymphocyte.mp or exp hemoglobins/ or h?emoglobin.mp | 3689693 | exp *hyponatremia/ or exp *hypernatremia/ or exp *hyperglycemia/ or exp *hypoglycemia/ or exp *insulin hypoglycemia/ or hypernatr?emi*.ti,ab or hyponatr?emi*.ti,ab or hyperglyc?emi*.ti,ab or hypoglyc?emi*.ti,ab or hyperkal?emi*.ti,ab or exp hyperkalemia/ or exp *methylmalonic acid/ or methylmalonic acid.ti,ab or exp *mineral/ or exp *mineral blood level/ or exp *mineral balance/ or exp *mineral deficiency/ or exp *mineral supplementation/ or exp *mineral intake/ or exp *mineral metabolism/ or mineral.ti,ab or exp *albumin/ or exp *serum albumin/ or exp *human albumin/ or exp *human serum albumin/ or exp *transthyretin/ or transthyretin.ti,ab or prealbumin.ti,ab or albumin.ti,ab or exp *transferrin/ or transferrin.ti,ab or exp *ferritin/ or exp *ferritin blood level/ or ferritin.ti,ab or exp *retinol-binding protein/ or retinol.ti,ab or RBP.ti,ab or exp *nitrogen/ or nitrogen.ti,ab or exp *nitrogen balance/ or exp *trace element/ or trace element.ti,ab or exp *selenium/ or exp *selenium deficiency/ or selenium.ti,ab or exp *iodide/ or iodide.ti,ab or exp *skin sensitivity/ or skin sensitivity.ti,ab or exp *C-reactive protein/ or c-reactive protein.ti,ab or CRP.ti,ab or exp *orosomucoid/ or orosomucoid.ti,ab or alpha-1 acid glycoprotein.ti,ab or exp *lymphocyte count/ or lymphocyte count.ti,ab or exp *hemoglobin/ or exp *glycosylated hemoglobin/ or h?emoglobin.ti,ab | 1115934 |
| 5 | exp celiac disease/ or c?eliac disease.mp or exp glutens/ or gluten.mp or exp food hypersensitivity/ or exp intestinal mucosa/ or exp wheat hypersensitivity/ or eDL/ or exp cholesterol, LDL/ or exp cholesterol, VLDL/ or exp cholesterol ester storage disease/ or exp cholesterol esters/ or exp cholesterol oxidase/ or exp cholesterol, dietary/ or cholesterol.mp or exp hyperlipidemias/ or hyperlipid?emi*.mp or exp hypertension/ or hypertensi*.mp or exp gastroenterology/ or gastroenterolog*.mp or exp intestinal diseases/ or intestin*.mp or enteropath*.mp | 1769635 | exp *celiac disease/ or c?eliac disease.ti,ab or exp *gluten/ or gluten.ti,ab or exp *cholesterol/ or exp *cholesterol intake/ or exp *cholesterol level/ or exp *cholesterol blood level/ or exp *total cholesterol level/ or exp * high density lipoprotein cholesterol/ or exp *high density lipoprotein cholesterol level/ or exp * low density lipoprotein cholesterol/ or exp *low density lipoprotein cholesterol level/ or exp *very low density lipoprotein cholesterol/ or exp *hyperlipidemia/ or hyperlipid?emi*.ti,ab or exp *lipid/ or exp *lipid level/ or exp *lipid blood level/ or cholesterol.ti,ab or lipid.ti,ab or exp *gastroenterology/ or gastr*.ti,ab or exp *enteropathy/ or enteropathy.ti,ab or intestin*.ti,ab | 2314643 |
| 6 | exp nutrition assessment/ or nutrition* assessment.mp or prognostic nutritional index.mp or prognostic inflammatory index.mp or subjective global assessment.mp or Birmingham nutrition risk score.mp or nutrition risk classification.mp or malnutrition screening tool.mp or simple screening tool.mp or malnutrition universal screening tool.mp or nutritional risk screening 2002.mp or NRS 2002.mp or nutritional risk screening.mp or exp nutritional status/ or controlling nutritional status.mp or CONUT.mp or Maastricht index.mp or nutritional risk index.mp or elderly nutritional indicators for geriatric malnutrition assessment.mp or ENIGMA.mp or swallowing.mp or exp deglutition/ or exp deglutition disorders/ or deglutition.mp or dysphagia.mp or speech language assessment.mp or SALT assessment.mp | 141188 | exp *nutritional status/ or exp *nutritional assessment/ or prognostic nutritional index.ti,ab or prognostic inflammatory index.ti,ab or subjective global assessment.ti,ab or Birmingham nutrition risk score.ti,ab or nutrition risk classification.ti,ab or mini nutritional assessment.ti,ab or malnutrition screening tool.ti,ab or simple screening tool.ti,ab or full nutritional assessment.ti,ab or malnutrition universal screening tool.ti,ab or nutritional risk screening 2002.ti,ab or NRS 2002.ti,ab or short nutrition assessment questionnaire.ti,ab or controlling nutritional status.ti,ab or CONUT.ti,ab or Maastricht index.ti,ab or nutritional risk index.ti,ab or elderly nutritional indicators for geriatric malnutrition assessment.ti,ab or ENIGMA.ti,ab or exp *swallowing/ or swallow*.ti,ab or exp *dysphagia/ or dysphagia.ti,ab or exp *nasogastric tube/ or nasogastric.ti,ab or speech language assessment.ti,ab or SALT assessment.ti,ab | 117942 |
| 9 | 1 or 2 or 3 or 4 or 5 or 6 | 14775283 | 1 or 2 or 3 or 4 or 5 or 6 | 8315294 |

COMPARISON: N/A

AND

OUTCOME:

| # | MEDLINE | Hits | EMBASE | Hits |
| --- | --- | --- | --- | --- |
| 1 | exp injury severity score/ or exp severity of illness score/ or exp trauma severity indices/ or severity.mp or mJOA.mp or nurick.mp or exp spinal fusion/ or cooper myelopathy scale.mp or CMS.mp or exp outcome assessment/ or exp quality of life/ or prolo score.mp or European myelopathy score.mp or exp spinal stenosis/ or exp intervertebral disc displacement/ or EMS.mp or Copenhagen Neck Functional Disability Scale.mp or CNFDS.mp | 832135 | exp *disease severity/ or exp *disability severity/ or exp *pain severity/ or exp *severity of illness index/ or exp disease severity assessment/ or exp *injury severity/ or exp *fatigue severity scale/ or severity.ti,ab or exp *follow up/ or follow* up.ti,ab or exp *surgery/ or mJOA.ti,ab or nurick.ti,ab or exp *Nurick grade/ or cooper myelopathy scale.ti,ab or CMS.ti,ab or exp *quality of life/ or quality of life.ti,ab or prolo score.ti,ab or exp *spine fusion/ or exp *intervertebral disk hernia/ or disk hernia.ti,ab or European myelopathy score.ti,ab or exp *vertebral canal stenosis/ or EMS.ti,ab or Copenhagen Neck Functional Disability Scale.ti,ab or CNFDS.ti,ab or exp *pain assessment/ or disability index.ti,ab | 4100509 |
| 2 | neurological deficit.mp or outcome.mp or neurological benefit.mp or exp encephalomyelitis, autoimmune, experimental/ or enchephal*.mp or exp pain/ or exp eye pain/ or exp chronic pain/ or exp facial pain/ or exp back pain/ or exp low back pain/ or exp nociceptive pain/ or exp neck pain/ or exp musculoskeletal pain/ or exp pain management/ or exp pain measurement/ or exp pain perception/ or exp pain, intractable/ or exp pain, postoperative/ or exp pain, procedural/ or exp shoulder pain/ or exp visceral pain/ or exp pain insensitivity, congenital/ or exp pain, referred/ or pain.mp or neuropath*.mp or exp neuralgia/ or exp spinal cord injuries/ or exp reperfusion injury/ or exp stroke/ | 2752459 | exp *neurological disease/ or neurological deficit.ti,ab or exp *pain/ or exp *neuropathic pain/ or exp *limb pain/ or exp *musculoskeletal pain/ or exp *visceral pain/ or exp *referred pain/ or exp *pleural pain/ or exp *postoperative pain/ or exp *procedural pain/ or exp *pain intensity or exp *pain measurement/ or exp *pain severity/ or exp *pain threshold/ or exp *posttraumatic pain/ or exp *spinal pain/ or exp *neck pain/ or exp *myofascial pain/ or exp *jaw pain/ or exp *inflammatory pain/ or exp *eye pain/ or exp face pain/ or exp *arm pain/ or exp *bone pain/ or exp *eyelid pain/ or exp *substernal pain/ or exp *wrist pain/ or exp *shoulder pain/ or pain.ti,ab or exp *neuropathy/ or exp *neuropathic pain/ or exp *brain function/ or neurological function.ti,ab or neuropath*.ti,ab | 2852780 |
| 3 | exp age of onset/ or onset.mp or exp treatment outcome/ or exp postoperative complications/ or outcome.mp or surgical results.mp or surgical management.mp or surgical success.mp or recovery.mp or exp vestibular function/ or exp ventricular function/ or exp respiratory function tests/ or exp recovery of function/ or exp pituitary function tests/ or exp liver function tests/ or exp kidney function tests/ or exp liver function tests/ or exp heart function tests/ or exp executive function/ or function.mp or neurological function.mp or exp patient readmission/ or readmission.mp or exp reoperation/ or reoperation.mp or exp hospitalization/ or hospitali?ation.mp or hospital*.mp | 6499214 | exp *adverse outcome/ or exp *adverse outcome pathway/ or exp *clinical outcome/ or exp *critical care outcome/ or exp *Glasgow outcome scale/ or exp *nursing outcome/ or exp *outcome assessment/ or exp *patient-reported outcome/ or exp *treatment outcome/ or outcome*.ti,ab or exp *late onset disorder/ or onset.ti,ab or surgical.ti,ab or exp *surgery/ or surgery.ti,ab or exp *anesthetic recovery/ or recovery.ti,ab or function.ti,ab or exp *function test/ or exp *motor function test/ or exp *muscle function/ or exp *musculoskeletal function/ or exp *parasympathetic function/ or exp *sympathetic function/ or exp *respiratory function/ or exp *spinal cord function/ or exp *vestibular function/ or exp *visual system function/ or exp *skin function/ or exp *urogenital system function and reproduction/ or exp *urinary tract function/ or exp *urethra function/ or exp *ureter function/ or exp *testis function/ or exp *sweat gland function/ or exp *sensorimotor function/ or exp *sexual function/ or exp *placental function/ or exp *pancreas function/ or exp *ovarian function/ or exp *ovary function/ or exp *nervous system function/ or exp *nerve function/ or exp *neuromuscular function/ or exp *mammary gland function/ or exp *liver function/ or exp *liver function test/ or exp *lacrimal gland function/ or exp *kidney function/ or exp *kidney function test/ or exp *intestine function/ or exp *intestine function disorder/ or exp *joint function/ or exp *hand function/ or exp *executive function/ or exp *executive function test/ or exp *endocrine function/ or exp *esophagus function/ or exp *cardiovascular function/ or exp *bladder function/ or exp *auditory system function/ or exp *adrenal cortex function/ or exp *adrenal function/ or exp *autonomic nervous system function/ or exp *cell function/ or exp *cognitive function test/ or exp *cardiopulmonary function/ or exp *brain function/ or exp *blood vessel function/ or exp *digestive system function disorder/ or exp *heart function/ or exp *mental function/ or exp *mental function assessment/ or exp *hospital readmission/ or readmission.ti,ab or exp *reoperation/ or reoperation.ti,ab or re-operation.ti,ab or exp *hospitalization/ or hospitali?ation.ti,ab or hospital*ti,ab | 2843340 |
| 4 | exp decompression, surgical/ or exp decompression sickness/ or exp spinal neoplasms/ or spinal cord decompression.mp or exp spinal cord compression/ or spinal cord compression.mp or spinal cord complications.mp or spinal cord surgery.mp | 55521 | exp *spinal cord decompression/ or exp *spinal cord compression/ or spinal cord decompression.ti,ab or spinal cord compression.ti,ab or exp *spinal cord surgery/ or spinal cord surgery.ti,ab | 16991 |
| 8 | 1 or 2 or 3 or 4 | 7476590 | 1 or 2 or 3 or 4 | 8030042 |

**Supplementary Data 3 – Data Extraction Form**

**Extraction Template**

*List of datapoints to be extracted:*

1. **Name of first author**
   1. Surname
   2. First initials
2. **Year of publication**
3. **Study Design**
   1. Prospective / Retrospective
   2. Cohort / Case Control / RCT
4. **Conflicts of interest (COI) in study**
   1. Yes (copy and paste COI statement) or no
5. **Study Sample characteristic**
   1. single or multi-centre
   2. number of patients
   3. country of patients
   4. start and end dates of study
6. **Patient demographics**
   1. age
   2. gender
   3. ethnicity
   4. socioeconomic status
   5. employment status
   6. history of diabetes
   7. weight
   8. height
   9. BMI
   10. other important patient characteristics (e.g. vitamin deficiencies, history of CVD)
   11. alcohol status
   12. smoking status
7. **Cohort type**
   1. pre-operative surgical, post-operative surgical or non-surgical
   2. heterogeneous group of cervical myelopathy patients or specific group of patients e.g. rheumatoid arthritis patients with cervical myelopathy
8. **Nutritional factor/intervention assessed and measurement scale** (e.g. weight, height, diabetes, blood glucose levels, hypoalbuminemia, vitamin deficiency, BMI, body fat percentage, alcohol status)
9. **Outcome(s) measured**
   1. Outcome(s) measured and scale (e.g. hospital readmission rates, infection rates, pain, functional outcome (e.g. Nurick score or mJOA), quality of life (eg. SF-36) gait analysis,)
   2. Overall, generalised outcome if stated (e.g. a percentage improvement in myelopathy)
   3. Overall means and standard deviations
   4. Means and standard deviations of relevant subgroup analyses
10. **Relationships between nutritional factor/intervention and outcome measures**
    1. Statistical tests and interpretation
11. **Funding body for paper**

**Supplementary Data 4 – Risk of Bias Assessment**

**Cohort studies – 50/57**

**
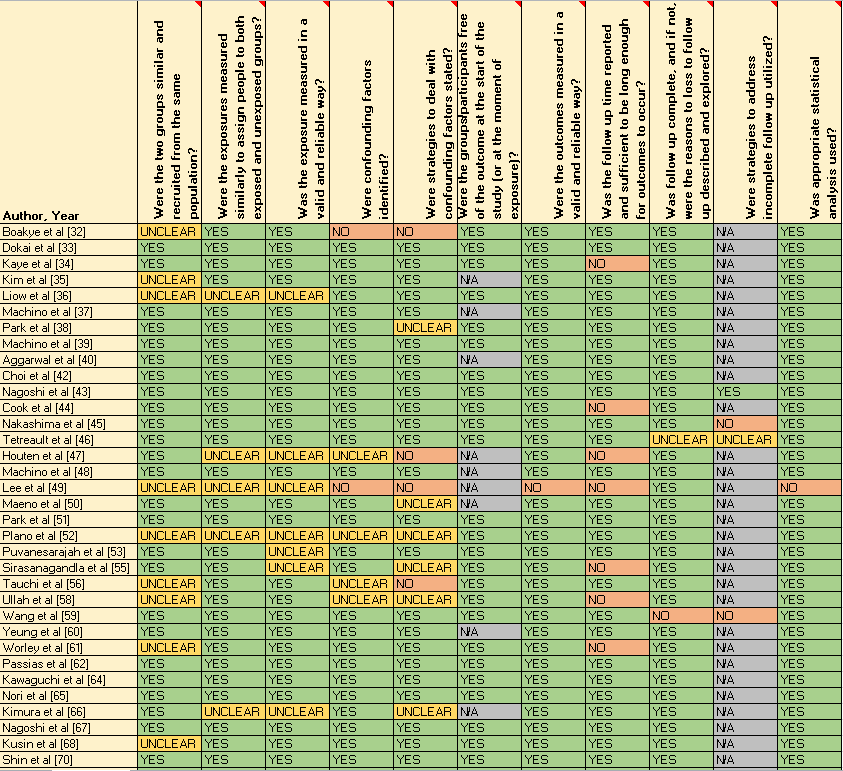
**

**
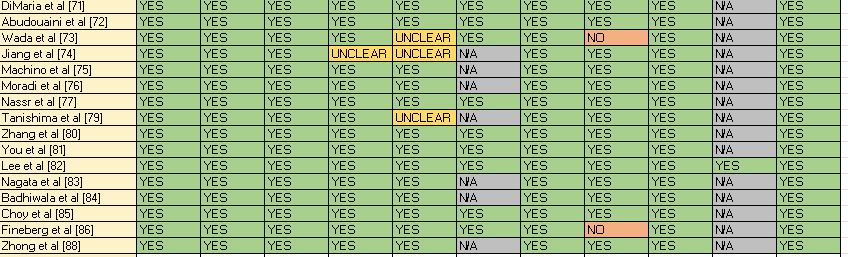
**

**Cross-sectional studies – 7/57**


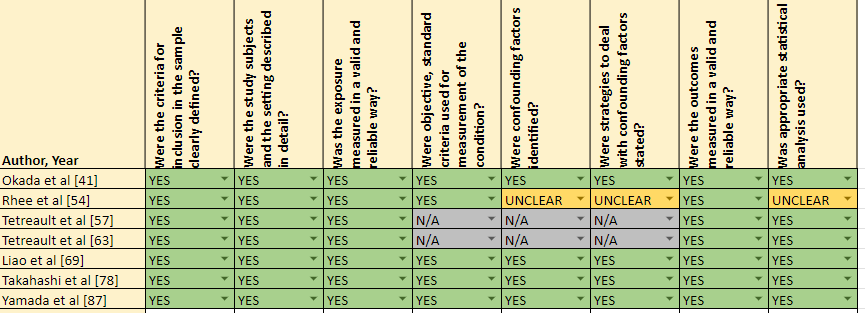

Supplement: Supplementary file 1 [file Data_Sheet_1.docx]
